# Cytotoxic Mechanism of Excess Polyamines Functions through Translational Repression of Specific Proteins Encoded by Polyamine Modulon

**DOI:** 10.3390/ijms21072406

**Published:** 2020-03-31

**Authors:** Akihiko Sakamoto, Junpei Sahara, Gota Kawai, Kaneyoshi Yamamoto, Akira Ishihama, Takeshi Uemura, Kazuei Igarashi, Keiko Kashiwagi, Yusuke Terui

**Affiliations:** 1 Faculty of Pharmacy, Chiba Institute of Science, Choshi, Chiba 288-0025, Japan; asakamoto@cis.ac.jp; 2 Faculty of Advanced Engineering, Chiba Institute of Technology, Chiba 275-0016, Japan; s1223113vc@s.chibakoudai.jp (J.S.); gota.kawai@p.chibakoudai.jp (G.K.); 3 Department of Frontier Bioscience, Hosei University, Tokyo 184-8584, Japan; kanyamam@hosei.ac.jp (K.Y.); aishiham@hosei.ac.jp (A.I.); 4 Amine Pharma Research Institute, Innovation Plaza at Chiba University, Chiba 260-0856, Japan; uemura@amine-pharma.com (T.U.); iga16077@gmail.com (K.I.); 5 Graduate School of Pharmaceutical Science, Chiba University, Chiba 260-0856, Japan

**Keywords:** polyamines, polyamine modulon, cytotoxicity, cell growth, cell viability, spermidine

## Abstract

Excessive accumulation of polyamines causes cytotoxicity, including inhibition of cell growth and a decrease in viability. We investigated the mechanism of cytotoxicity caused by spermidine accumulation under various conditions using an *Escherichia coli* strain deficient in spermidine acetyltransferase (SAT), a key catabolic enzyme in controlling polyamine levels. Due to the excessive accumulation of polyamines by the addition of exogenous spermidine to the growth medium, cell growth and viability were markedly decreased through translational repression of specific proteins [RMF (ribosome modulation factor) and Fis (rRNA transcription factor) etc.] encoded by members of polyamine modulon, which are essential for cell growth and viability. In particular, synthesis of proteins that have unusual locations of the Shine–Dalgarno (SD) sequence in their mRNAs was inhibited. In order to elucidate the molecular mechanism of cytotoxicity by the excessive accumulation of spermidine, the spermidine-dependent structural change of the bulged-out region in the mRNA at the initiation site of the *rmf* mRNA was examined using NMR analysis. It was suggested that the structure of the mRNA bulged-out region is affected by excess spermidine, so the SD sequence of the *rmf* mRNA cannot approach initiation codon AUG.

## 1. Introduction

Polyamines, which are thought to be present at mM concentrations in most growing cells, are essential for cell growth and viability [1]. Polyamines, such as putrescine (PUT) and spermidine (SPD), are distributed ubiquitously across all domains of life. Intracellular polyamine concentrations are strictly regulated through biosynthesis, degradation, uptake, and excretion, since depletion or excessive accumulation of polyamines causes cell growth inhibition and lower cell viability [2,3]. In mammalian cells, the main polyamines consist of SPD and spermine (SPM). Cytotoxic products, such as acrolein, hydrogen peroxide, aminoaldehydes, and ammonia, are produced by the oxidation of SPM during polyamine catabolism. Among them, acrolein is a highly toxic unsaturated aldehyde and has been shown to inhibit cell growth [4]. However, in many mesophilic bacteria, the main polyamines consist of PUT and SPD, but not SPM [5], so acrolein cannot be produced by the oxidation of polyamines due to the lack of a polyamine oxidase gene. Therefore, the cytotoxic mechanism that results from accumulation of polyamines in bacteria has not been characterized in detail.

Because polyamines exist mostly as polyamine-RNA complexes in cells [6], their effects on cell growth and viability are presumed to be caused by the changes in RNA functions. We previously reported that polyamines stimulate general protein synthesis in vitro, increase the fidelity of translation [7,8], and induce the assembly of 30S ribosomal subunits in vivo [9,10]. In addition, we reported that polyamines enhanced synthesis of the 20 specific proteins important for cell growth and viability at the level of translation in *Escherichia coli* [11,12,13]. The genes encoding these 20 different proteins were termed as the “polyamine modulon”. There are three different mechanisms underlying stimulation of protein synthesis from the genes encoded by polyamine modulon. Firstly, polyamines stimulate protein synthesis when the Shine–Dalgarno (SD) sequence in the mRNA is obscure or is distant from initiation codon AUG. Polyamines cause structural changes of a region of the SD sequence and initiation codon AUG, then facilitate formation of the initiation complex. In particular, the structural change of the bulged-out region of a hairpin structure of 5′-untranslated regions by polyamines is important for polyamine stimulation of these protein syntheses [14]. This is the case for *cpxR*, *emrR*, *fecI* (σ^18^), *fis*, *hns*, *oppA*, *rmf*, *rpoE* (σ^24^), *rpoN* (σ^54^), *rpoZ* (ω), *soxR*, and *stpA*. Secondly, polyamines enhance the inefficient initiation codon UUG- or GUG-dependent fMet-tRNA binding to ribosomes. This is the case for *cra*, *cya*, *frr*, *gshA*, *spoT*, and *uvrY*. Thirdly, polyamines stimulate read-through of amber codon UAG on ribosome-associated *rpoS* (σ^38^) mRNA by facilitating the binding of Gln-tRNA^supE^, or stimulating a +1 frameshift at the 26th UGA codon of the *prfB* mRNA encoding RF2.

The intracellular content of polyamines, such as PUT and SPD, in *E. coli* is elaborately regulated by biosynthesis, degradation, and transport. Among these, SPD acetyltransferase is a key catabolic enzyme in controlling polyamine concentrations. This enzyme is not required for cell growth in *E. coli* cells, but is involved in the responses to a variety of chemical and physical stresses [15,16]. It has also been reported that polyamine catabolism is a core metabolic response to several stresses [17]. These reports indicate that polyamine metabolism is important for stress response, while we previously reported that SPD toxicity is increased in response to excessive accumulation of SPD when SPD acetyltransferase is lacking [18]. In an SPD acetyltransferase-disrupted mutant, the protein synthesis of the RMF, a ribosome modulation factor involved in the formation of 100S dimers from 70S ribosomes, RpoS, a σ^38^ sigma factor of the RNA polymerase involved in the expression of genes at the stationary phase, and the OmpC, an outer membrane protein C which is a cation-selective porin protein in the outer membrane, were particularly inhibited. Cell viability was decreased in the triple *rmf*, *rpoS*, *ompC* mutant at the stationary phase. However, when SPD accumulated in cells, cell toxicity occurred more rapidly than expected [19]. These results suggested that other factors are involved in cell toxicity in addition to these three proteins.

In this study, we carried out experiments to study in detail the cytotoxic mechanisms of excessive accumulation of SPD under various stress conditions using an *E. coli* CAG2242 strain deficient in SPD acetyltransferase. We found that due to the excessive accumulation of polyamines by the addition of SPD to the medium, cell growth and viability were decreased through the translational repression of specific proteins encoded by members of polyamine modulon. In particular, the syntheses of proteins encoded by *cpxR*, *emrR*, *fecI* (σ^18^), *fis*, *oppA*, *rmf*, *rpoE* (σ^24^), *rpoN* (σ^54^), *rpoZ* (ω), *soxR*, and *stpA*, which have unusual locations of the SD sequence in their mRNAs, were inhibited. Among these genes, we focused on the *rmf* gene whose translation was significantly repressed by SPD. In order to elucidate a mechanism of inhibition of cell growth and viability resulting from excessive accumulation of polyamines, the selective structural change of the bulged-out region in the mRNA at the initiation site of the *rmf* mRNA by SPD was investigated using NMR analysis. It was shown that cell growth and viability were decreased by excess polyamines through translational repression of specific proteins encoded by polyamine modulon, whose SD sequence in the mRNA is located more distally than most genes from initiation codon AUG.

## 2. Results

### 2.1. Correlation between the Decrease in Cell Growth and Translation of Proteins Encoded by Polyamine Modulon in Response to Excess Spermidine

As shown in Table 1, 20 members of the *E. coli* polyamine modulon were classified according to three mechanisms for polyamine stimulation of protein synthesis under various conditions, and thirteen members were identified as transcriptional factors. The proteins encoded by polyamine modulon strongly contribute to cell growth at the logarithmic phase, and to cell viability at the stationary phase.

**Figure 1:**
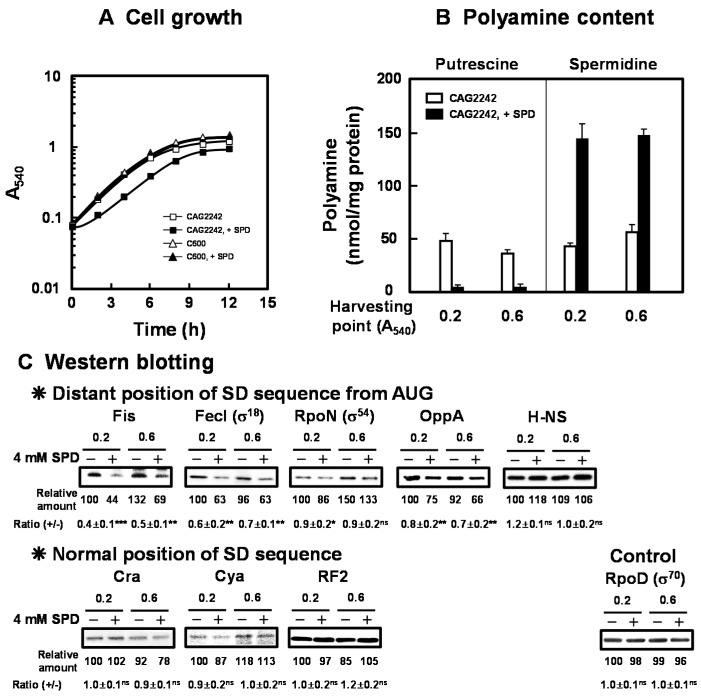
Inhibition of cell growth of *E. coli* CAG2242 by excess SPD. Cell growth of *E. coli* CAG2242 cells with or without 4 mM SPD. Values are means of triplicate determinations. Error bars are within the symbol size (**A**). Polyamine contents in cells harvested at A_540_ of 0.2 and 0.6 were measured as described under “Materials and Methods” (**B**). The levels of Fis, FecI (σ^18^), RpoN (σ^54^), OppA, H-NS, Cra, Cya, and RF2 were measured together with a control protein, RpoD (σ^70^), by Western blotting (**C**). Values are means ± S.E. of triplicate determinations. Student’s *t*-test was performed for the values obtained in the presence of SPD versus in the absence of SPD. ns, *p* ≥ 0.05; *, *p* < 0.05; **, *p* < 0.01; ***, *p* < 0.001.

We previously reported that *E. coli* CAG2242, an SPD acetyltransferase-disrupted mutant, showed accumulation of SPD and subsequent decrease in cell viability in the late stationary phase when cells were cultured in the presence of SPD (0.5 mM to 2 mM) [18]. The overall change in cell growth was not altered by 2 mM SPD. To clarify the mechanisms underlying cytotoxicity of excessive accumulation of SPD in detail, *E. coli* CAG2242 was cultured in the presence of 4 mM SPD. As shown in Figure 1A, growth of *E. coli* CAG2242 was significantly inhibited by 4 mM SPD. The growth of parental *E. coli* cell C600 was not influenced by 4 mM SPD (Figure 1A). Polyamine contents in the cells grown with or without SPD were then measured at the early (A_540_ = 0.2) and the late logarithmic phase (A_540_ = 0.6) (Figure 1B). Intracellular water space of *E. coli* was estimated to be 2.9 µL of cell volume/mg of protein [31]. In addition, most SPD (about 90% of the total) forms complexes with RNA non-covalently in cells [6]. When *E. coli* CAG2242 cells were cultured without SPD, a normal amount of SPD (approximately 10 to 15 mM) was detected in the cells. When the cells were cultured with SPD, accumulation of SPD was observed from the early logarithmic phase. The amount of accumulated SPD in the cells was approximately 3- to 5-fold of that in the cells grown without SPD, whereas the level of PUT was decreased by the addition of SPD. These results suggested that the concentration of SPD in the cells was approximately 45 mM, and a high level of SPD as the sole polyamine in cells inhibited growth of CAG2242. It was confirmed that the synthesis of proteins encoded by members of polyamine modulon at the early to late logarithmic phase was significantly decreased by excess SPD, judging from the level of proteins (Figure 1C). In particular, the synthesis of proteins essential for cell growth (Fis, FecI (σ^18^), RpoN (σ^54^), and OppA, but not H-NS) encoded by members of polyamine modulon that have the position of the SD sequence distant from initiation codon AUG in their mRNAs was significantly inhibited by excess SPD. However, the synthesis of the proteins (Cra, Cya, and RF2) encoded by members of polyamine modulon that have a normally positioned SD sequence in their mRNAs was not inhibited significantly by excess SPD. Levels of mRNAs encoded by polyamine modulon were not influenced by excess SPD (Figure S1). These results indicate that the synthesis of proteins encoded by polyamine modulon is inhibited by excess SPD at the translational level. As a control, the effect of SPD on RpoD (σ^70^ transcriptional factor, a major σ factor) synthesis was examined, and excess SPD did not reduce RpoD synthesis significantly. Considering the protein synthesized at the logarithmic phase, synthesis of H-NS was not influenced by excess SPD. H-NS is a member of nucleoid-associated DNA-binding proteins, including StpA. H-NS and StpA share similar functions, but are regulated differently by growth temperature. It was reported that the level of H-NS decreased and that of StpA increased at 42 °C compared to those protein levels at 37 °C [32]. Thus, we next determined whether the syntheses of the proteins encoded by members of polyamine modulon were altered by excess SPD under various growth conditions, such as elevated growth temperature.

**Figure 2:**
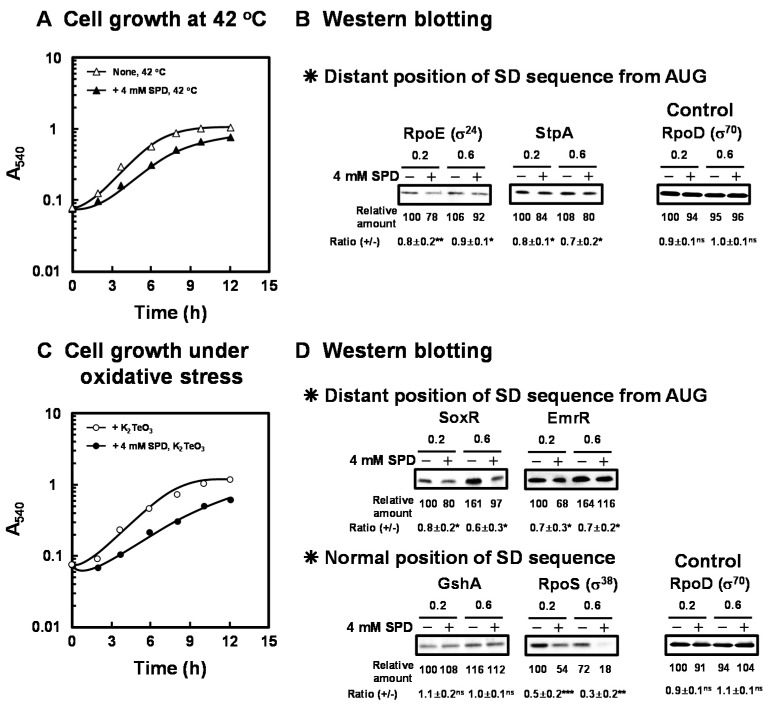
Inhibition of cell growth of *E. coli* CAG2242 by excess SPD under heat shock stress and oxidative stress conditions. *E. coli* CAG2242 cells were cultured as described in the legend of Figure 1 at 42 °C (**A**) and in the presence of 0.6 µM K_2_TeO_3_ (**C**) with or without 4 mM SPD. Values are means of triplicate determinations. Error bars are within the symbol size. The levels of RpoE (σ^24^), StpA, SoxR, EmrR, and GshA were measured together with a control protein, RpoD (σ^70^), by Western blotting (**B**,**D**). Values are means ± S.E. of triplicate determinations. Student’s *t*-test was performed for the values obtained in the presence of SPD versus in the absence of SPD. ns, *p* ≥ 0.05; *, *p* < 0.05; **, *p* < 0.01; ***, *p* < 0.001.

As shown in Figure 2A, the reduction of growth rate of *E. coli* CAG2242 by 4 mM SPD was not altered at 42 °C compared to 37 °C. However, the lag phase of growth of *E. coli* CAG2242 in the presence of 4 mM SPD at 42 °C was prolonged, and the yield was less at 12 h. Therefore, growth of *E. coli* CAG2242 in the presence of 4 mM SPD at 42 °C was slightly slower than at 37 °C. These results indicate that cell toxicity caused by excess SPD was slightly increased at the elevated temperature. The synthesis of the proteins essential for cell growth under heat shock conditions [RpoE (σ^24^), StpA] encoded by members of polyamine modulon that have the position of the SD sequence distant from initiation codon AUG in their mRNAs was slightly but significantly inhibited by excess SPD (Figure 2B).

As shown in Figure 2C, when *E. coli* CAG2242 was cultured in the presence of K_2_TeO_3_, an inducer of oxidative stress, especially superoxide anion [33], the degree of cell toxicity by excess SPD for cell growth was greater than that in the cells cultured in the absence of K_2_TeO_3_. The growth rate reduced, the lag phase was significantly prolonged, and the final yield also reduced to a large degree in the presence of 4 mM SPD under oxidative stress. Furthermore, growth of *E. coli* CAG2242 in the presence of 4 mM SPD under oxidative stress was slower than under heat shock stress. Under these conditions, the syntheses of SoxR (redox systems) and EmrR were significantly inhibited by excess SPD (Figure 2D). These members of polyamine modulon have the position of the SD sequence distant from initiation codon AUG in their mRNAs. In contrast, the synthesis of GshA which has a normal position of the SD sequence was not influenced by excess SPD. However, the level of RpoS which has a normal position of the SD sequence was decreased by SPD under these conditions. It has also been reported that the stringent response factor, ppGpp, which is required for long-term survival [34], negatively regulates *rpoS* transcription [35], and synthesis of ppGpp is enhanced by polyamines [36]. Moreover, it has been reported that *rpoE* and *rpoS* control transcription of polyamine catabolic enzymes (*patA* and *patD*) [17]. Thus, these proteins encoded by polyamine modulon are at least partially involved in the decrease in cell growth under various stress conditions.

### 2.2. Correlation between the Decrease in Cell Viability and Translation of Proteins Encoded by Polyamine Modulon in Response to Excess Spermidine

**Figure 3:**
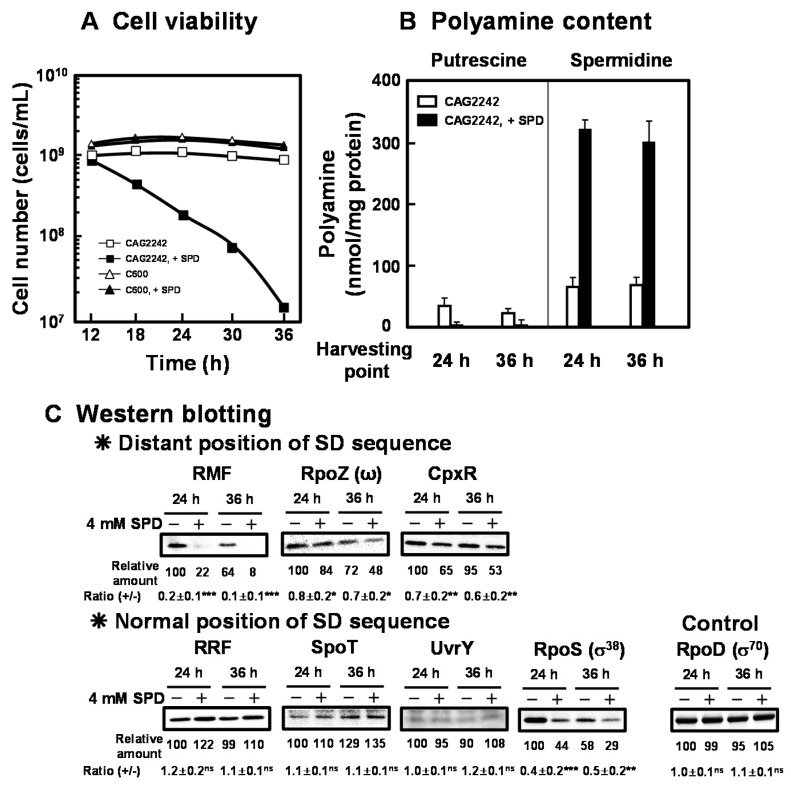
Inhibition of cell viability of *E. coli* CAG2242 by excess SPD. *E. coli* CAG2242 cells were cultured as described in the legend of Figure 1. Cell viability was determined by counting colony numbers in aliquots of the culture grown on LB-containing 1.5% agar plates at 37 °C. Values are means of triplicate determinations. Error bars are within the symbol size (**A**). Polyamine contents in the cells harvested at A_540_ of 24 h and 36 h were measured as described under “Materials and Methods” (**B**). The levels of RMF, RpoZ (ω), CpxR, RRF, SpoT, UvrY, and RpoS (σ^38^) were measured together with a control protein, RpoD (σ^70^), by Western blotting (**C**). Values are means ± S.E. of triplicate determinations. Student’s *t*-test was performed for the values obtained in the presence of SPD versus in the absence of SPD. ns, *p* ≥ 0.05; *, *p* < 0.05; **, *p* < 0.01; ***, *p* < 0.001.

As shown in Figure 3A, cell viability of *E. coli* CAG2242 with or without SPD was examined. Due to the accumulation of SPD, cell viability of *E. coli* CAG2242 was decreased greatly at the stationary phase, but that of *E. coli* C600 was not altered (Figure 3A). Polyamine contents in cells grown with or without SPD were then measured at the stationary phase (24 and 36 h) (Figure 3B). When *E. coli* CAG2242 cells were grown without SPD, a small amount of SPD was detected in the cells. When the cells were grown with SPD, overaccumulated SPD was observed at the stationary phase. The accumulated SPD in the cells was approximately 6 times higher than that in the cells grown without SPD, whereas the level of PUT decreased over time. These results suggest that the concentration of SPD in the cells that could decrease cell viability of *E. coli* CAG2242 was approximately 90 mM. The synthesis of the proteins necessary for cell viability (RMF, RpoZ, and CpxR) encoded by members of polyamine modulon that have the position of the SD sequence distant from initiation codon AUG in their mRNAs was significantly inhibited by excess SPD (Figure 3C). However, the synthesis of the proteins (RRF, SpoT, UvrY, but not RpoS) encoded by members of polyamine modulon that have a normally positioned SD sequence were not influenced by excess SPD. The level of RpoS was decreased by SPD at both the logarithmic and the stationary phase in parallel. Thus, it is possible that excess polyamines reduce cell viability greatly by regulating synthesis of both ppGpp and RpoS, which inhibit RNA and protein synthesis.

### 2.3. Recovery of Cell Growth and Viability by Transformation of the Gene Encoding Spermidine Acetyltransferase

**Figure 4:**
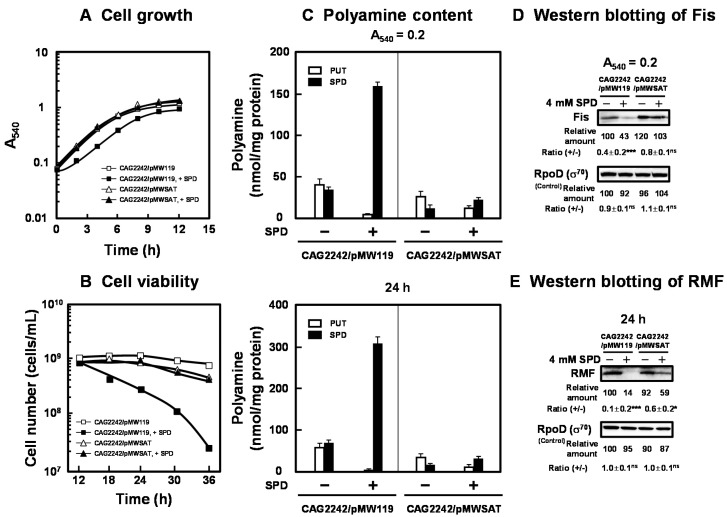
Recovery of cell growth and viability by SPD acetyltransferase. *E. coli* CAG2242 cells carrying pMWSAT or pMW119 (vector) were cultured with or without 4 mM SPD (**A**). Cell viability was determined by counting colony numbers in aliquots of the culture grown on LB-containing 1.5% agar plates at 37 °C (**B**). Polyamine contents in the cells harvested at A_540_ of 0.2 and 24 h were measured as described under “Materials and Methods” (**C**). The levels of RMF and Fis were measured together with a control protein, RpoD (σ^70^), by Western blotting (**D**,**E**). Values are means ± S.E. of triplicate determinations. Student’s *t*-test was performed for the values obtained in the presence of SPD versus in the absence of SPD. ns, *p* ≥ 0.05; *, *p* < 0.05; ***, *p* < 0.001.

The effect of SPD acetyltransferase on cell growth and viability was assessed. As shown in Figure 4A,B, the transformant of *E. coli* CAG2242 with pMWSAT encoding the gene for SPD acetyltransferase recovered cell growth and viability in the presence of excess SPD. Polyamine contents in the cells grown with or without SPD were then measured at the logarithmic and stationary phases (Figure 4C). The level of SPD in cells was markedly decreased when pMWSAT was transformed. We previously reported that cell growth and viability of *E. coli* MA261, a polyamine-requiring mutant, was reduced by depletion of polyamines. Under the depletion of polyamines, the transformant of *E. coli* MA261 with *fis* and *rmf* genes with modifications in the position of the SD sequence to the normal position recovered cell growth and viability [20,25]. Thus, the synthesis of Fis, a global regulator of transcription of some growth-related genes, including genes for rRNA and tRNA, was examined at the logarithmic phase, since Fis is an essential protein for cell growth. In addition, the synthesis of RMF was examined at the stationary phase, since RMF is an important protein for cell viability. With pMWSAT, Fis and RMF were synthesized efficiently even in the presence of SPD (Figure 4D). By the addition of excess SPD, cell growth and viability were enhanced in parallel with the increase in the levels of Fis and RMF (Figure 4A). These results indicate that cell growth and viability are reduced by excess polyamines through translational repression of the specific proteins encoded by polyamine modulon whose SD sequence in the mRNA is located distant from initiation codon AUG.

### 2.4. Analysis by NMR of the Structural Change of the Bulged-out Region at the Initiation Site of the *rmf* mRNA by SPD

**Figure 5:**
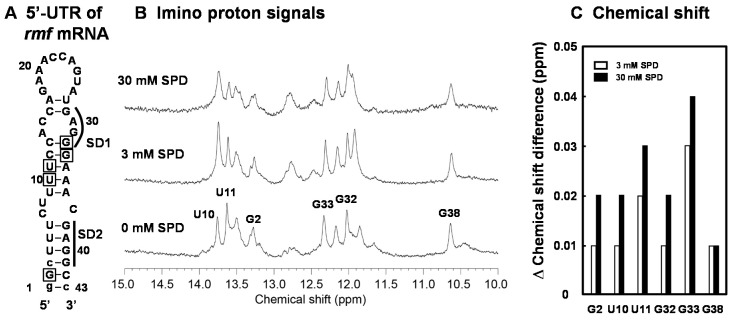
Analysis of the structural change of the bulged-out region at the initiation site of the *rmf* mRNA by SPD using NMR. The predicted secondary structure of the designed RNA stem-loop for the initiation site of the *rmf* mRNA. The RNA consists of the -1 to -41 nucleotides of the 5′-untranslated region from initiation codon AUG. The numbers show the position from the 5′-end of the nucleotide sequence. The bases shown in lowercase were necessary for in vitro transcription (**A**). NMR spectra in the imino proton region in the absence and presence of SPD (**B**). The chemical shift difference (ppm) induced by SPD is shown in (**C**). The residues whose chemical shift changes are shown in Figure 5C are indicated by a square in Figure 5A.

We have previously shown that the structural change of the bulged-out region of the double-stranded RNA induced by SPD is strongly involved in polyamine stimulation of specific protein syntheses [23]. In order to elucidate a cytotoxic mechanism resulting from excessive accumulation of SPD in detail, the structural change of the initiation region of the *rmf* mRNA (RMF-WT RNA) by SPD was evaluated by NMR analysis. In Figure 5A, the predicted secondary structure (43-mer nucleotides) of the initiation region of the *rmf* mRNA was constructed based on the predicted secondary structure of the region by vsfold [37]. The 1D imino proton spectrum of the RMF-WT RNA showed that the overall secondary structure was not changed, but local structures were affected by addition of 3 mM or 30 mM SPD (Figure 5B). The chemical shift change of assigned imino proton signals for G2, U10, U11, G32, G33, and G38 by the addition of SPD are shown in Figure 5C. In general, the chemical shift changes induced by 30 mM SPD were greater than those induced by 3 mM SPD indicating that more SPD molecules bind to the RNA in the presence of excess amount of SPD. This result suggests that the structure including the bulged-out region in the mRNA became more structured in the presence of excess SPD, so that the SD sequence of the *rmf* mRNA cannot approach initiation codon AUG.

## 3. Discussion

In this study, we tried to clarify the cytotoxic mechanism resulting from excess SPD in *E. coli*. It is thought that cell toxicity is mainly caused by reactive oxygen species [38]. However, acrolein produced mainly from SPM is more toxic than reactive oxygen species [39,40]. SPM is normally absent in most mesophilic bacteria such as *E. coli*, *Helicobacter pylori*, and *Pseudomonas* species [41,42]. Therefore, the cytotoxic mechanism resulting from accumulation of polyamines in bacteria has not been characterized in detail. We examined the cytotoxic mechanism of excessive accumulation of SPD using an *E. coli* strain deficient in SPD acetyltransferase, which regulates polyamine concentrations.

Interestingly, we found that cell growth and viability were reduced by excess polyamines through translational repression of the specific proteins encoded by polyamine modulon whose SD sequence in the mRNA is located distant from initiation codon AUG. The synthesis of the specific proteins encoded by polyamine modulon that have a normal position of the SD sequence in the mRNA was not influenced by excess polyamines at the translational level. Among the identified polyamine modulon proteins, the synthesis of Fis and RMF was strongly inhibited by accumulated SPD (Figure 2 and Figure 4). There was no change in intracellular magnesium with or without SPD (data not shown). The transformant of *E. coli* CAG2242 with pMWSAT recovered cell growth and viability with an increase in the translational level of Fis and RMF in the presence of excess SPD (Figure 4). Thus, the decreases in the proteins encoded by polyamine modulon by the accumulated SPD are probably one of the reasons for the decrease in cell growth and viability. In particular, when the cell viability in the event of polyamine deficiency and of excess polyamines was compared, that of excess polyamines decreased more rapidly [25]. Since synthesis of RMF was inhibited by the accumulated SPD, cell viability is strongly affected, and excess polyamines are more toxic to cells than polyamine deficiency. These results confirm that polyamines contribute to cell growth and viability of *E. coli* under various stress conditions.

Several reports showed that the effect of SPD is mainly caused by stabilization of the bulged-out region of the double-stranded RNA, whose structure is not stabilized by Mg^2+^ [14,43]. We have previously reported that two candidate SD sequences exist in the nucleotide sequence of the *rmf* mRNA [25]. The first SD sequence (SD1) located at 11 nucleotides upstream of initiation codon AUG forms the bulged-out region, and the second SD sequence (SD2) located at 2 nucleotides upstream of initiation codon AUG forms the double-stranded RNA (Figure 5A). When the position of the SD1 of the *rmf* mRNA was shifted to the more common position located at 8 nucleotides upstream of initiation codon AUG, the degree of polyamine stimulation was reduced, although the level of the RMF protein was increased in the cells cultured without polyamines. When the SD2 was modified together with the SD1, the level of the RMF protein was markedly increased in the cells cultured without polyamines because of the elimination of the SD2, which forms the double-stranded RNA. On the other hand, the level of the RMF protein of transformant CAG2242 with the modified RMF (SD1) was inhibited similarly to the wild type under the accumulation of SPD (data not shown). These results suggest that a mechanism for stimulation of RMF synthesis by the optimal concentration of polyamines is different from that for inhibition of RMF synthesis by excess polyamines. The effect may be caused by the difference in the binding site of the *rmf* mRNA by SPD. Therefore, we carried out experiments to clarify how different concentrations of SPD influence the structure of the bulged-out region of the double-stranded RNA. The chemical shift changes of imino proton signals in the *rmf* mRNA induced by excess SPD were greater than the ones induced by the optimal concentration of SPD for cell growth and viability. The overall change and chemical shift (3 to 30 mM) in RNA structures of two mutants were not altered compared to the *rmf* wild-type RNA. Thus, we speculated that synthesis of the RMF at the translational level was reduced due to the inhibition of initiation complex formation by excess SPD; structural stability of stems with bulge-outs are regulated by SPD in the low concentration range, but, in the presence of excess SPD, the stability is too high. However, it is not clear why SPD preferentially binds to the polyamine modulon mRNA whose SD sequence is located distant from initiation codon AUG. In addition, the degree of inhibition of protein synthesis by excess SPD was different. These results strongly suggest that the optimal concentration of polyamines is necessary for cell growth and viability. To clarify the mechanism of inhibition of protein synthesis in detail, we investigated the RNA secondary structure of the initiation region of these mRNAs using the vsfold program. The bulged-out structure tended to exist in the polyamine modulon mRNA whose SD sequence is located distant from initiation codon AUG. However, it was difficult to characterize the difference in structure of the initiation site of these mRNAs. Further analyses, such as detailed NMR analysis, and the culture conditions may be required. However, the results obtained in this report strongly suggest that the optimal concentration of polyamines is necessary for optimal cell growth and viability.

## 4. Materials and Methods

### 4.1. Bacterial Strains, Culture Conditions, and Plasmid

*E. coli* CAG2242 (*speG*, *supE44*, *hsdR*, *thi*, *thr*, *leu*, *lacY1*, *tonA21*) was kindly supplied by Dr. E. W. Gerner. The cells were cultured overnight at 37 °C in a modified Luria–Bertani (LB) medium (10 g of tryptone, 5 g of yeast extract, and 10 g of NaCl per liter) containing tetracycline (30 µg/mL). Eighty µl of bacterial culture were transferred to 10 mL of an M9 medium [44] containing 1% potassium lactate, 2 µg/mL thiamine, and 100 µg/mL each of leucine and threonine and grown till A_540_ reached 1.0. Then, *E. coli* cells were cultured at the A_540_ of 0.1, and growth was monitored at 37 °C by measuring A_540_ in the M9 medium containing 1% potassium lactate, 2 µg/mL thiamine, and 100 µg/mL each of leucine and threonine with or without 4 mM spermidine. Cell viability was determined by counting colony numbers in aliquots of the culture grown on LB-containing 1.5% agar plates at 37 °C.

pMWSAT was constructed as described previously [18]. *E. coli* CAG2242 transformed with pMWSAT or pMW119 was grown in the presence of ampicillin (100 µg/mL).

### 4.2. Measurement of Polyamine Contents

Polyamines in *E. coli* were extracted by treatment of the cells with 10% trichloroacetic acid (TCA) with occasional shaking. Polyamine contents were determined by high-performance liquid chromatography as described previously [45]. Protein content was determined by the method of Bradford [46].

### 4.3. Western Blot Analysis

Western blot analysis was performed by the method described in [47] using horseradish peroxidase-conjugated anti-rabbit IgG (GE Healthcare Bio-Sciences) as a secondary antibody and ECL Western blotting reagents (GE Healthcare Bio-Sciences). Antibodies against the proteins encoded by polyamine modulon were prepared as described previously [21,24,26,27,48]. The level of protein on the blot was quantified with a LAS-3000 luminescent image analyzer (Fuji Film).

### 4.4. Prediction of the Secondary Structure of RNA

RNA secondary structure of the RMF-WT RNA was predicted by the vsfold program [37].

### 4.5. Preparation of the RNA

The RMF-WT RNA which contains the −1 to −41 nucleotides of the 5′-untranslated region from initiation codon AUG was prepared by the in vitro transcription method with the T7 RNA polymerase. The DNA template for transcription, antisense strands, and an 18-mer sense strand for the T7 promoter (5′-CTAATACGACTCACTATA-3′) were purchased from Hokkaido System Science Co., Ltd. Transcripts were purified by 12% polyacrylamide gel electrophoresis (PAGE) in the denatured condition (7 M urea). After PAGE purification, the samples were extracted by water and subjected to ethanol precipitation followed by ultracentrifugation by Vivaspin 2 (Sartorius, 3000 MWCO PES). RNA samples were dissolved in a 20 mM sodium phosphate buffer (pH 6.5) containing 50 mM NaCl, 1 mM MgCl_2_, and 5% D_2_O. Concentration of the RMF-WT sample for NMR measurements was 0.08 mM.

### 4.6. NMR Measurements and Signal Assignment

NMR spectra were measured using AVANCE-600 spectrometers (Bruker BioSpin K.K.). Spectra were recorded at probe temperatures of 288 K. The water signal was suppressed by the jump-and-return pulse [49]. NMR spectra were analyzed with Topspin (Bruker BioSpin K.K.), and the imino proton signals were assigned by the conventional method based on NOESY spectra.

## Figures and Tables

**Table 1:** Three mechanisms of polyamine stimulation of the protein synthesis encoded by polyamine modulon.

Mechanism	Increase in Cell Growth	Increase in Cell Growth under Heat Shock Stress	Increase in Cell Growth and Viability under Oxidative Stress	Increase in Cell Viability
(1) Long distance between the SD sequence and initiation codon AUG	Fis [20] FecI (σ^18^) [20] RpoN (σ^54^) [21] OppA [22] H-NS [21]	RpoE (σ^24^) [23] StpA [23]	SoxR [24] EmrR [24]	RMF [25] RpoZ (ω) [26] CpxR [27]
(2) Initiation on an inefficient initiation codon (normal position of the SD sequence from AUG)	Cra [21] Cya [28]		GshA [24]	RRF [27] SpoT [26] UvrY [27]
(3) Suppression or +1 frameshifting on a nonsense codon (normal position of the SD sequence from AUG)	RF2 [29]		RpoS (σ^38^) [30]	RpoS (σ^38^) [30]

## Supplementary Materials

Supplementary materials are available online at https://www.mdpi.com/1422-0067/21/7/2406/s1.

## Abbreviations

NMR	nuclear magnetic resonance
ppGpp	guanosine 5′-diphosphate 3′-diphosphate
PUT	putrescine
SAT	spermidine acetyltransferase
SD	Shine–Dalgarno
SPD	spermidine
SPM	spermine
