# Supplementary material for: Cytotoxic Mechanism of Excess Polyamines Functions through Translational Repression of Specific Proteins Encoded by Polyamine Modulon
Source: Int J Mol Sci. 2020 Mar 31;21(7):2406. doi: 10.3390/ijms21072406 (PMC7177335; doi:10.3390/ijms21072406)
Supplement: Supplementary file 1 [file ijms-21-02406-s001.pdf]

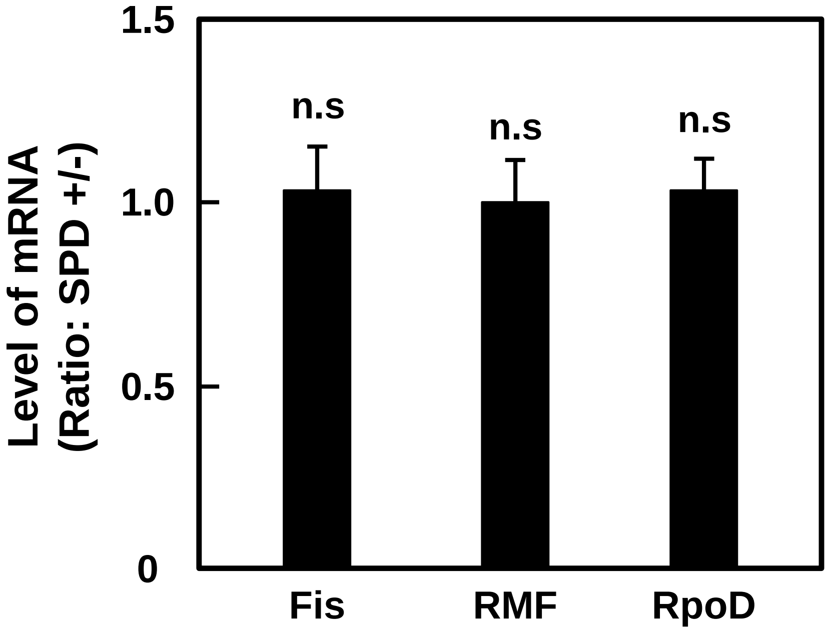

Supplementary Figure 1. Levels of *fis, rmf* and *rpoD* mRNAs. Levels of mRNAs were measured by qPCR. Values are means ± S. E. of triplicate determinations. Student's *t* test was performed for the value obtained in the presence of 4 mM SPD versus in the absence of SPD. ns, $p \geq 0.05$.

Materials and Methods

Quantitative real time PCR

Total RNA was extracted from CAG2242 cultured with or without 4 mM SPD by NucleoSpin®RNA (TaKaRa). Complementary DNA was synthesized using a cDNA synthesis kit (ReverTra-Plus-™; TOYOBO), and a Quantitative real time PCR was performed using the SYBR Green master mix and Applied Biosystems® 7500 real time PCR system.

Primers used for qPCR.

Fis,

Forward 5′-GTTCGAACAACGCGTAAATTCTGACG-3′

Reverse 5′-GGTGTATTGCATCACCATGTCCAACA-3′

RMF,

Forward 5′-CAGTATGAGGGAAACGAGGCATGAAGAGAC-3′

Reverse 5′-AATCAGGCCATTACTACCCTGTCCGCCATG-3′

RpoD,

Forward 5′-ATGGAGCAAAACCCGCAGTCACAGCTGAAA-3′

Reverse 5′-AGCATCAGATCATCGGCATCCGGTGCTTCT-3′
